# Hypertriglyceridemia: a too long unfairly neglected major cardiovascular risk factor

**DOI:** 10.1186/s12933-014-0159-y

**Published:** 2014-12-04

**Authors:** Alexander Tenenbaum, Robert Klempfner, Enrique Z Fisman

**Affiliations:** Cardiac Rehabilitation Institute, Sheba Medical Center, 52621 Tel-Hashomer, Israel; Sackler Faculty of Medicine, Tel-Aviv University, 69978 Tel-Aviv, Israel; Cardiovascular Diabetology Research Foundation, 58484 Holon, Israel

**Keywords:** Cardiovascular risk, Cholesterol, Fibrates, Hypertriglyceridemia, Insulin resistance, Metabolic syndrome, Obesity, Statins, Triglycerides, Type 2 diabetes

## Abstract

The existence of an independent association between elevated triglyceride (TG) levels, cardiovascular (CV) risk and mortality has been largely controversial. The main difficulty in isolating the effect of hypertriglyceridemia on CV risk is the fact that elevated triglyceride levels are commonly associated with concomitant changes in high density lipoprotein (HDL), low density lipoprotein (LDL) and other lipoproteins**.** As a result of this problem and in disregard of the real biological role of TG, its significance as a plausible therapeutic target was unfoundedly underestimated for many years. However, taking epidemiological data together, both moderate and severe hypertriglyceridaemia are associated with a substantially increased long term total mortality and CV risk. Plasma TG levels partially reflect the concentration of the triglyceride-carrying lipoproteins (TRL): very low density lipoprotein (VLDL), chylomicrons and their remnants. Furthermore, hypertriglyceridemia commonly leads to reduction in HDL and increase in atherogenic small dense LDL levels. TG may also stimulate atherogenesis by mechanisms, such excessive free fatty acids (FFA) release, production of proinflammatory cytokines, fibrinogen, coagulation factors and impairment of fibrinolysis. Genetic studies strongly support hypertriglyceridemia and high concentrations of TRL as causal risk factors for CV disease. The most common forms of hypertriglyceridemia are related to overweight and sedentary life style, which in turn lead to insulin resistance, metabolic syndrome (MS) and type 2 diabetes mellitus (T2DM). Intensive lifestyle therapy is the main initial treatment of hypertriglyceridemia. Statins are a cornerstone of the modern lipids-modifying therapy. If the primary goal is to lower TG levels, fibrates (bezafibrate and fenofibrate for monotherapy, and in combination with statin; gemfibrozil only for monotherapy) could be the preferable drugs. Also ezetimibe has mild positive effects in lowering TG. Initial experience with en ezetimibe/fibrates combination seems promising. The recently released IMPROVE-IT Trial is the first to prove that adding a non-statin drug (ezetimibe) to a statin lowers the risk of future CV events. In conclusion, the classical clinical paradigm of lipids-modifying treatment should be changed and high TG should be recognized as an important target for therapy in their own right. Hypertriglyceridemia should be treated.

## Introduction

The independent association between elevated triglycerides (TG), cardiovascular (CV) risk and mortality has been largely controversial [1,2]. The main difficulty in isolating the effect of hypertriglyceridemia on CV is the fact that elevated TG levels are commonly associated with concomitant changes in high density lipoprotein (HDL), low density lipoprotein (LDL) and other lipoproteins**.** Although the majority of studies found a substantial direct association between TG and adverse outcomes [3-12], this association sometimes became nonsignificant after multivariate adjustment including other lipids and weight-related variables [13-16]. For example, in the largest till now meta-analysis [13], TG were associated with an increased risk of coronary artery disease (CAD) after adjustment for age and sex, but this association was abolished following an additional adjustment for HDL and non-HDL cholesterol. Correlations with lower HDL cholesterol led to studies in which the authors mathematically “adjusted” for these relationships, suggesting that the HDL values could be invoked as more likely contributors to risk than the TG themselves. As a result of these mathematical over-adjustment exercises and in disregard of the real biological role of TG, the significance of hypertriglyceridemia as a plausible therapeutic target was unfoundedly underestimated for many years. However, epidemiology can be a poor guide to clinical decisions and gives us only limited insight into the mechanisms of atherogenesis and their relative importance in this process. On the other hand, classification of elevated TG as a major CV risk factor is clinically important since it determines whether high TG should be a target for therapy in their own right.

### Hypertriglyceridemia and CV events

Serum TG are routinely measured under fasting conditions to obtain more stable concentrations and to enable the physician to calculate LDL cholesterol levels. In addition, hypertriglyceridemia and postprandial lipidemia may affect the measurement of HDL cholesterol and therefore the calculation of non-HDL cholesterol.

The NCEP ATP III arbitrarily divided fasting serum TG into four different classes [17]. Classification of serum TG levels greater than 150 mg/dl (1.7 mmol/liter) as elevated is mainly based on large prospective observational studies. However, the exact level at which serum TG start to confer risk or become a marker for CV disease is unknown, but it may be even lower than 150 mg/dl (1.7 mmol/liter) [18]. Serum TG are higher in men and increase with age in both sexes [19].

Very high TG (correspondent to severe hypertriglyceridemia) are defined as serum TG > or = 500 mg/dl [3], whereas the Endocrine Society Clinical Practice Guideline [20] labeled as very severe hypertriglyceridemia serum TG > or = 2000 mg/dl. The common view was that severe and very severe hypertriglyceridemia increase the risk for pancreatitis, whereas mild or moderate hypertriglyceridemia may be a risk factor for CV disease [20]. In patients with very high TG levels – i.e. more than 25 mmol/L - and patients with the familial chylomicronemia syndrome the risk for atherosclerosis is attenuated, perhaps because their plasma lipoprotein particles are too large to enter into the arterial intima [21] and [22]. However, there are many indications that there is an increased risk of CV disease in the marked or severe hypertriglyceridemia (fasting triglyceride concentration exceeding 5.6 mmol/L and 11.2 mmol/L) as well [23,24]. Moreover, even in the prominent old study of Assmann et al. [21] which emphasized a J-shape for TG cardiovascular risk, TG above 800 mg/dl were still associated with significantly greater risk than TG less than 200 mg/dl, albeit it was decreased in comparison to the 400–799 mg/dl levels.

Prospective studies have indicated that, compared with fasting levels, nonfasting serum TG levels may be a better or similar predictor of CV events in the general population [25-29]. In a number of studies using standardized meals, greater CV risk was found to be associated with increased hypertriglyceridemia [27,28].

The serum TG concentration is often more strongly correlated with future CAD incidence in univariate analysis than is serum cholesterol. However, in multiple logistic regression analysis, particularly when HDL cholesterol is included, the strength of the apparent independent relationship between TG and CAD incidence is weakened often to the point of insignificance in individual trials. The erosion of the relationship between TG and CAD incidence when HDL is included in multiple logistic regression analysis is to some extent an artifact of the greater biological variation of TG concentrations compared with HDL cholesterol. When allowance is made, TG can have more predictive power than HDL [30]. Taking epidemiological data together, both moderate and severe hypertriglyceridaemia are associated with a substantially increased long term total mortality and CV risk.

### Triglycerides and atherogenesis

Currently LDL is considered as the major atherogenic lipoprotein; however other lipoproteins size is of key importance in determining whether particles can penetrate the arterial wall. The plasma TG level represents in part the concentration of the TRL: VLDL, chylomicrons and their remnants. Although chylomicrons and probably VLDL are both too large to penetrate the arterial wall, their remnants are small enough to do this, and have been demonstrated in human and animal atherosclerotic plaques [31].

Physiologically TG are the densest form of calories and serve as an important source of energy. Dietary TG are assembled in the gut into chylomicrons. Their interaction with lipoprotein lipase (LpL) located on the luminal surface of capillary endothelial cells leads to liberation of free fatty acids from TG; free fatty acids are able to traverse cell membranes. Only 50% of chylomicron’s TG is estimated to be lost in this process, and the remainder of the lipoprotein, called a chylomicron remnant, contains lipids such as cholesteryl esters, retinylesters, and apoB-48 [20].

VLDL particles are the main TG carrier in the circulation, being produced by the liver, whereas the VLDL TG content is derived from a variety of substrates including lipoprotein TG and FFA. VLDL TG lose FFA by the action of LpL as well, leading to production of VLDL remnants, also referred to as intermediate-density lipoproteins (IDL), and eventually to conversion to LDL. The concentration of VLDL cholesterol and apolipoprotein B (apoB) is at least 10 times higher than the corresponding chylomicron concentration, even after consumption of a large amount of fat [32-35]. These lipoproteins contain at least as much cholesterol per particle as does LDL. TG itself is not a component of arterial plaque, but cholesterol within TG -rich particles contributes to plaque development [36,37].

VLDL can be divided into large, TG -rich VLDL1 and small, dense VLDL2. VLDL1 has a higher TG content and exhibit abundant apolipoprotein CIII (apoCIII) and apolipoprotein E [38,39].

An increase in TG-rich lipoproteins is commonly associated with a reduction in HDL and an increase in small dense LDL levels. Hypertriglyceridemia stimulates the enzymatic activity of cholesteryl ester transfer protein (CETP), which facilitates the transfer of TG from TG -rich lipoproteins to HDL and LDL in exchange for cholesteryl esters [40]. This leads to an increase in TG content of HDL and LDL. TG -enriched HDL particles are subject to increased catabolism; consequently, they have a short plasma half-life. TG-enriched LDL particles undergo subsequent hydrolysis via LpL or hepatic lipase, thereby reducing LDL particle size. In addition, the difference in metabolic fate between VLDL1 and VLDL2 may also account for the increased formation of small dense LDL. Kinetic data show that large TG -rich VLDL1 particles yield small dense LDL whereas smaller and denser VLDL2 particles are metabolized to normal sized LDL [41].

TG may also stimulate atherogenesis by other mechanisms, which include the production of proinflammatory cytokines, fibrinogen and coagulation factors and impairment of fibrinolysis. Therefore, their roles in atherogenesis have a basic biological plausibility.

### Hypertriglyceridemia as a major component of atherogenic dyslipidemia related to insulin resistance, MS and T2DM

Hypertriglyceridemia results from increased TG production, or reduced TG catabolism, or both. Drugs such as bile acid resins, estrogens, isotretinoin and steroids; marked alcohol and fat ingestion in a susceptible patient; or conditions such as poorly controlled diabetes or pregnancy can result in high triglyceride levels [42,43].

One of the reason for hypertriglyceridemia is alcohol consumption. Alcohol intake increases hepatic fatty acid synthesis and decreases fatty acid oxidation, with a net effect to stimulate hepatic VLDL TG secretion. The effects of alcohol are dose-dependent [44,45]. However the most common forms of hypertriglyceridemia are related to overweight and sedentary life style which leads to insulin resistance. This setting of hypertriglyceridemia is typical for MS and T2DM. The increase in TG production may be due to excess FFA returning to the liver, particularly in the setting of visceral obesity and insulin resistance, and increased de novo TG production due to hyperinsulinemia [46-48]. In hypertriglyceridemia, more VLDL particles, as measured by apoB, and larger and more TG- and apoC-III-enriched lipoproteins are found [49-51]. Hepatic insulin resistance may contribute to a high production rate of VLDL because insulin reduces apoB synthesis and VLDL secretion in the liver [52,53]. Although insulin resistance is associated with high triglycerides, VLDL and TG concentrations can be similar in patients with widely divergent insulin sensitivity [54,55].

Hypertriglyceridemia, as one of the components of the MS, is closely related to a constellation of metabolic risk factors including a central distribution of adiposity or visceral obesity, insulin resistance, impaired glucose tolerance, hypertension, and high TG and/or low HDL-C, associated with an atherogenic, procoagulant, and proinflammatory state [56-64].

One could thought that TG are not directly involved in the development of atherosclerotic lesions because FFA released from TG by lipoprotein lipase (LpL) act either as an active energy source or stored energy reserve. However, there are several plausible mechanisms by which FFA might cause CV disease and death [65-71]. Furthermore, an increase in plasma FFA leads to endothelial activation, inflammation and thrombosis which may initiate early vascular abnormalities that promote atherosclerosis [72-77]. Elevation of plasma FFA, in addition to producing peripheral and hepatic insulin resistance, also activates the proinflammatory NFκB pathway [73-84] resulting in increased hepatic expression of several proinflammatory cytokines including TNF-α, IL1-β, IL6, matrix metalloproteinases and an increase in circulating MCP-1 [85-89], supporting the notion that FFA is an important link between hypertriglyceridemia and the development of inflammatory changes [90-99]. Moreover, elevated plasma FFA levels, via producing insulin resistance and hyperinsulinemia, promote a state of increased tendency for thrombosis and decreased ability to fibrinolysis. Together, this substantially increases the risk for acute atherothrombotic events [100-105].

Therefore, elevated FFA are not only an independent risk factor for the development of T2DM, but also give rise to metabolic derangements in organs such as the liver and pancreas. Hypertriglyceridemia, FFAs overload and lipid accumulation in non-adipose tissues influence both insulin action and insulin secretion and are frequently associated with IR and the development of T2DM [106-109]. However the role of FFAs extends beyond their ability to induce or exacerbate insulin resistance: they may contribute directly to the deterioration of beta cell function that accompanies the development of diabetes [109-113]. Both acute stimulatory and long-term detrimental effects of FFAs overloading on pancreatic beta-cell have long been recognized. Chronic exposure of the pancreatic beta-cell to FFA results in desensitization and suppression of secretion (lipotoxicity) as a consequence of TG accumulation in the islets of Langerhans [109].

**Figure 1:**
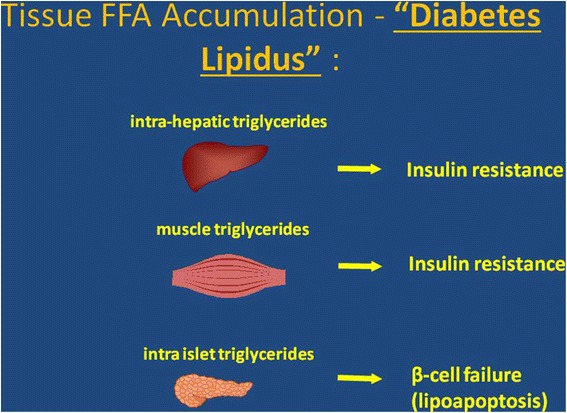
**Hypertriglyceridemia, FFAs overload and lipid accumulation in nonadipose tissues (so called lipotoxicity) are the key events in the pathogenesis of T2DM, mainly via insulin resistance and pancreatic beta-cell failure.**

Since evidence indicates that multiple aberrations in lipids metabolism play a pivotal role in the pathophysiology of diabetes, it was suggested to drop the adjective “mellitus” from diabetes and then to consider the introduction of a new adjective “lipidus” or “lipomellitus” [114] (Figure 1).

### TG and HDL

Raised TG concentrations are strongly associated with low concentrations of HDL cholesterol, and the past 25 years have been dominated by HDL research, with less focus on TG. The hypothesis that HDL is protective against atherosclerosis was supported by a series of animal studies in the 1980s and 1990s. Badimon and colleagues [115] infused HDL into rabbits and reported inhibition of atherosclerosis. Rubin and colleagues [116] showed that mice overexpressing the major HDL protein apolipoprotein A-I (apoA-I) are protected from atherosclerosis. Viral overexpression of apoA-I in mice with pre-existing atherosclerosis resulted in regression of pre-existing atherosclerotic disease [117]. These preclinical data matched the epidemiological data and strongly reinforced the HDL hypothesis, making HDL a major target for novel therapeutic approaches to decrease atherosclerosis.

Consequently, HDL has long been regarded as the “good” lipoprotein because epidemiological and clinical studies have identified an inverse association between HDL concentration and CV disease [118,119]. The most important antiatherogenic function of HDL is reverse cholesterol transport [120]. HDL also exhibits other potential cardioprotective functions such as anti-oxidative, anti-inflammatory and endothelium-dependent vasodilatory effects [121-123].

However, a failing initial experience with CETP inhibitors has been most problematic for the HDL hypothesis [124-126]. Moreover, reports from several randomized clinical trials of HDL-raising drugs have failed to show a reduction in CV events. Particularly, two recent trials of niacin (using extended-release niacin; AIM-HIGH [127] and HPS2-THRIVE [128]) were done on the background of statin therapy and were primarily designed to show the benefit of the HDL-raising effects of niacin. Neither trial met its primary endpoint and niacin failed to reduce cardiovascular events in both trials. Based on this, extended-release niacin added to a statin in patients with reasonably controlled LDL-C concentrations does not confer a cardiovascular benefit despite an increase in HDL-C concentrations. As a result, niacin at present should not be considered a therapeutic option for raising HDL-C concentrations.

In accordance with the HDL function hypothesis, it is not HDL cholesterol itself that has a causal relation to atheroprotection, but rather HDL function, which cannot be reliably estimated through the simple measurement of HDL-C [129,130].

Interesting lessons could be derived from the Bezafibrate Infarction Prevention (BIP) study with bezafibrate and basically low HDL in all patients: despite of a significant HDL raising, the overall benefits were non-significant. However, the benefit of bezafibrate in the subgroup of patients with high TG levels was extremely impressive.

Additional challenges to the HDL hypothesis are driven by data derived from human genetic studies and randomized controlled trials. Taken together, genetic studies strongly support the theory that high concentrations of TG-rich lipoproteins or remnant cholesterol are causal risk factors for cardiovascular disease and all-cause mortality [2,131-138], and that low HDL cholesterol is probably an innocent bystander. Low HDL cholesterol might merely be a long-term marker of raised TG and remnant cholesterol. Alternatively, HDL cholesterol might be a marker of cardiovascular health but is non-causal in the atherogenesis [130].

### Management of hypertriglyceridemia

Intensive lifestyle therapy, including dietary counseling to achieve appropriate diet composition, physical activity, and a program to achieve weight reduction in overweight and obese individuals are the main initial treatment of hypertriglyceridemia and described elsewhere [20,139-141].

Elevated levels of TG (and TG -rich lipoproteins) are increasingly being recognized as treatment targets to lower CV risk in certain patient subgroups, including individuals receiving statins – a cornerstone of the modern lipids-modifying therapy. The choice of statin should depend on the needs of the individual patient. In this context, the potential benefits of pitavastatin versus other statins in the treatment of patients with dyslipidemia and insulin resistance, metabolic syndrome or type 2 diabetes should be emphasized [142-144]. Moreover, although some statins are associated with increased haemoglobin A1C levels in patients receiving intensive but not moderate therapy, pitavastatin has demonstrated neutral or even favourable effects on glucose control in patients with and without T2DM or MS [145,146]. However, intensive statin therapy with the most potent statins (atorvastatin, rosuvastatin and even pitavastatin) does not completely eliminate the residual cardiovascular risk associated with high TG.

At present the number of drug classes (fibrates, niacin, n-3 fatty acids, CETP –inhibitors, ezetimibe, glitazars, etc.) alone or in combination with statins have been considered as treatment options in patients with moderate to severe TG levels. However, many of these agents are currently under serious concerns: niacin after the negative AIM HIGH study and HPS-2 THRIVE trial results [127,128]. CETP inhibitors and glitazars are still in controversial developments and not available for clinical use. Supplemental n-3 polyunsaturated fatty acids (PUFAs), mainly eicosapentaenoic acid and docosahexaenoic acid, are well known to reduce hypertriglyceridemia [147]. In addition to hypotriglyceridemic effects, omega-3 fatty acids may attenuate inflammation, improve endothelial function and reduce thrombus formation [148,149]. However, recent clinical outcome trials with have failed to show significant CV benefits in high risk subjects [150-152].

Ezetimibe inhibits intestinal cholesterol absorption and primarily lowers LDL cholesterol via the Niemann-Pick C1- Like 1 protein. Ezetimibe has slight positive effects in lowering plasma fasting TG (8%) [153]. In addition, ezetimibe reduces the cholesterol content of both fasting and postprandial TG -rich lipoproteins, thereby lowering the concentrations of atherogenic remnant particles [154]. Initial experience of ezetimibe/fibrates combination seems promising [155,156]. The recently released IMPROVE-IT (IMProved Reduction of Outcomes: Vytorin Efficacy International Trial) is the first to prove that adding a non-statin drug (ezetimibe) to a statin (simvastatin) lowers the risk of future CV events. Compared to patients with CAD on simvastatin plus a placebo, those on both simvastatin and ezetimibe, had a 6.4% lower risk of all CV events, a 14% lower risk of all heart attacks, a 14% lower risk of stroke, and a 21% lower risk of ischemic stroke. Deaths from CV disease were statistically the same in both groups. Patients were followed an average of approximately six years, and some as long as 8.5 years. Approximately 2 patients out of every 100 patients treated for 7 years avoided a heart attack or stroke. The Number Needed to Treat was = 50 [157]. This result represents a strong evidence-based support for the concept of the benefits of an appropriate statin/non-statin combination therapy.

Fibrates enhance the oxidation of fatty acids in liver and muscle and reduce the rate of hepatic lipogenesis, thereby reducing secretion of VLDL TG. The increased uptake of t TG -derived fatty acids in muscle cells results from an increase in LpL activity in adjacent capillaries and a decrease in the apolipoprotein CIII (apo CIII) concentration mediated transcriptionally by peroxisome proliferator activated receptor (PPAR) alpha. The decrease in apolipoprotein CIII reduces the inhibition of LpL activity. The enhanced catabolism of VLDL generates surface remnants, which are transferred to HDL. HDL concentrations are further augmented by an increase in PPAR alpha - mediated transcription of apoAI) and apo AII. Ultimately, the rate of HDL-mediated reverse cholesterol transport may increase. Fibrates activate PPAR alpha, which binds to a PPAR alpha response element in conjunction with the retinoid X receptor. Other effects of fibrates include an increase in the size of LDL particles, increased removal of LDL, and a reduction in the levels of plasminogen activator inhibitor type I [158-162].

From a clinical point of view, in all available 5 randomized control trials the beneficial effects of major fibrates (gemfibrozil, fenofibrate, bezafibrate) were clearly demonstrated and were highly significant in patients with hypertriglyceridemia [163-168].

In a meta-analysis of five dyslipidemic subgroups totaling 4726 patients, a 35% relative risk reduction in CV events was observed compared with a non- significant 6% reduction in those without dyslipidemia [169]. Meta-analysis performed in a so called "general population" [170] reflecting a blend of effects in patients with and without atherogenic dyslipidemia - a mean diluted effect of fibrate therapy was reduced, producing only 13% RR reduction for coronary events (p < 0.0001). Therefore, in patients with high triglycerides, fibrates - either as monotherapy or combined with statins - are consistently associated with reduced risk of cardiovascular events [171,172]. Therefore, if the primary goal is to lower TG levels, fibrates (bezafibrate and fenofibrate for monotherapy and combination with statin; gemfibrozil only for monotherapy) now are the preferable drugs [173,174].

## Conclusions

Taking epidemiological data together, both moderate and severe hypertriglyceridaemia are associated with a substantially increased long term total mortality and CV disease risk. The plasma TG level represents in part the concentration of the TRL: VLDL, chylomicrons and their remnants. TG may also stimulate atherogenesis by other mechanisms, which include the production of proinflammatory cytokines, fibrinogen and coagulation factors and impairment of fibrinolysis. The most common forms of hypertriglyceridemia are related to overweight and sedentary life style which leads to insulin resistance and typical for MS and T2DM. Therefore, the role of hypertriglyceridemia in atherogenesis has a multifactorial biological plausibility. Also genetic studies strongly support the theory that hypertriglyceridemia and high concentrations of TRL are causal risk factors for CV disease and mortality.

Intensive lifestyle therapy is the main initial treatment of hypertriglyceridemia. If the primary goal is to lower TG levels, fibrates (bezafibrate and fenofibrate for monotherapy and combination with statin; gemfibrozil only for monotherapy) now are the preferable drugs. Finally, the clinical paradigm of lipids-modifying treatment should be changed and high TG should be recognized as an important target for therapy in their own right. Hypertriglyceridemia should be treated.

## Abbreviations

apoA-I: Apolipoprotein A-I
apoB: Apolipoprotein B
apo CIII: Apolipoprotein CIII
CAD: Coronary artery disease
CETP: Cholesteryl ester transfer protein
CV: Cardiovascular
FFA: Free fatty acids
HDL: High density lipoprotein
LDL: Low density lipoprotein
LpL: Lipoprotein lipase
MS: Metabolic syndrome
PPAR: Peroxisome proliferator activated receptor
TG: Triglyceride, triglycerides
TRL: Triglyceride-carrying lipoproteins
T2DM: Type 2 diabetes mellitus
